# Relationship Between Frank’s Sign and Cardiovascular Disease: An Autopsy-Based Study

**DOI:** 10.7759/cureus.91756

**Published:** 2025-09-07

**Authors:** Bashirah Mohd Nor, Wan Mohammad Hafiz Wan Razali, Faridah Mohd Nor

**Affiliations:** 1 Forensic Unit, Department of Pathology, Faculty of Medicine, Universiti Kebangsaan Malaysia, Kuala Lumpur, MYS; 2 Department of Forensic Pathology, Faculty of Medicine, Universiti Teknologi MARA, Sungai Buloh, MYS; 3 Department of Forensic Pathology, Hospital Al-Sultan Abdullah, Universiti Teknologi MARA, Puncak Alam, MYS

**Keywords:** autopsy, cardiovascular death, coronary artery atherosclerosis, earlobe crease, frank’s sign

## Abstract

Frank's sign, defined as a diagonal crease on the earlobe extending from the tragus to the outer edge, has been proposed as a potential external marker for cardiovascular (CV) mortality. While previous autopsy studies have linked it to fatal outcomes of coronary artery disease, this study was designed to compare its prevalence in CV versus non-CV deaths.

A cross-sectional study of autopsy cases was conducted at Hospital Canselor Tuanku Muhriz, Universiti Kebangsaan Malaysia, from September 2023 to December 2024, examining 51 CV deaths and 51 matched non-CV deaths. The presence and severity of Frank's sign in both ears were assessed from photographs and correlated with critical coronary artery stenosis. Data were analyzed using IBM SPSS Statistics for Windows, Version 29.0 (Released 2022; IBM Corp., Armonk, NY, USA). Descriptive statistics were applied, chi-square tests were used to assess associations, and multivariable logistic regression controlled for confounders.

The study population (N = 102) had a mean age of 46.5 years and was predominantly male (83.3%). The analysis revealed a significantly higher prevalence of Frank's sign in CV deaths compared to non-CV deaths (χ² = 18.21, p < 0.001). Furthermore, a strong association was found between the sign and critical atherosclerotic coronary artery occlusion at autopsy (χ² = 19.79, p < 0.001). Even after adjusting for age, sex, and ethnicity, Frank's sign remained a strong independent predictor of critical coronary occlusion, with an adjusted odds ratio of 7.77 (p < 0.001). The diagnostic performance, measured by Youden's index, was moderate, at 0.39 for predicting CV death and 0.42 for predicting critical occlusion, indicating a moderate discriminative ability.

In summary, this study confirms a significant association between Frank's sign and CV mortality due to coronary artery atherosclerosis occlusion, suggesting its potential utility as a supportive, external clinical marker.

## Introduction

Cardiovascular diseases (CVDs) are the leading cause of mortality worldwide, with an estimated 17.9 million deaths annually. Ischemic heart disease alone accounts for approximately 16% of all deaths, underscoring its dominant role in global mortality patterns [1,2]. The burden is particularly pronounced in Southeast Asia, where recent estimates reported 36.8 million cases of CVD and 1.66 million deaths in 2021, with Malaysia recording the highest prevalence and mortality rates in the region [3]. National statistics confirm that ischemic heart disease has remained the leading cause of death in Malaysia, particularly among males [4].

Sudden cardiac death (SCD) represents a critical subset of cardiovascular (CV) mortality. The World Health Organization provides one of the most widely recognized definitions, describing SCD as an unexpected death occurring within one hour of symptom onset, if witnessed, or within 24 hours of last being seen alive and symptom-free, if unwitnessed [5]. Adabag et al. further defined SCD as an unexpected natural death from cardiac causes occurring within one hour of symptom onset, if witnessed, or within 24 hours of last being seen alive, if unwitnessed. Most cases result from arrhythmias secondary to acute myocardial infarction, with coronary heart disease underlying approximately 80%; in nearly half of patients, SCD is the first manifestation of heart disease [6]. Pathological definitions typically recognize ≥70%-75% coronary artery luminal obstruction as clinically significant in SCD [7,8].

Frank’s sign, a diagonal earlobe crease first described in 1973, has been investigated as a possible external marker of atherosclerotic CVD. Frank’s sign is characterized by a visible wrinkle, sulcus, or deep groove with an obscured base. Proposed mechanisms include microvascular changes and accelerated aging [9-11]. Clinical studies and systematic reviews have demonstrated an association with coronary artery disease, particularly in cases that involve both earlobes, although the sign lacks sufficient diagnostic accuracy to serve as a stand-alone test [12,13]. Evidence from autopsy studies further supports this association, with Kirkham et al. reporting a strong correlation between Frank’s sign and fatal CVD [14-16]. It has been reported that, while both Frank’s sign and coronary artery disease become more common with increasing age, their association remains significant [17]. In summary, this study aimed to assess whether Frank’s sign is associated with CV deaths and with the severity of coronary artery stenosis at autopsy in a Malaysian medicolegal population.

## Materials and methods

Study design

This cross-sectional observational study was conducted at the Forensic Unit, Hospital Canselor Tuanku Muhriz, Kuala Lumpur, Malaysia, between September 2023 and December 2024. The primary objective was to determine the prevalence of Frank’s sign in autopsy cases and to compare its occurrence between CV and non-CV deaths. A secondary objective was to evaluate the association between Frank’s sign and critical atherosclerotic coronary artery occlusion, regardless of the cause of death.

Eligible cases included Malaysian decedents aged 18-70 years, with bilateral ear photographs available at autopsy. Exclusion criteria were the presence of earrings, ear deformities, congenital cardiac anomalies, or advanced decomposition, charring, or skeletal remains. CV deaths in our study specifically referred to deaths due to coronary artery atherosclerosis, ischemic heart disease, or myocardial infarction, as confirmed at autopsy. Other CV causes, such as myocarditis, cardiomyopathy, and valvular disease, were excluded from our definition, in order to focus on coronary artery disease, which has the strongest reported association with Frank’s sign. 

**Figure 1:**
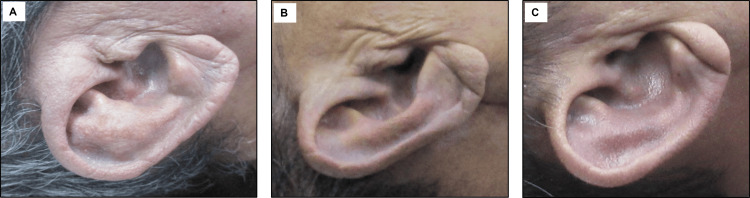
Clinical photographs of positive Frank’s sign (A) Earlobe wrinkling, (B) presence of visible sulcus, and (C) deep earlobe sulcus

Demographic variables (age, sex, and ethnicity) and standardized ear photographs were collected, and Frank’s sign was assessed from the photographs. Assessment was performed bilaterally and categorized as positive Frank's sign if any of the following were present: wrinkling, visible sulcus, or deep sulcus (Figure 1). Coronary artery stenosis due to atherosclerosis was classified as either non-critical (<75% stenosis) or critical (≥75% stenosis) in accordance with Sheppard’s pathological criteria [8].

Study sample

The study sample comprised medicolegal cases that underwent postmortem examination at the Forensic Unit. Cases were categorized into CV and non-CV deaths based on autopsy findings. The CV deaths group included deaths attributed to coronary atherosclerosis, ischemic heart disease, or myocardial infarction, while the non-CV group included both natural and unnatural manners of death. Natural deaths consisted of pulmonary thromboembolism, pneumonia, ruptured berry aneurysm, and epilepsy, whereas unnatural deaths consisted of traumatic fatalities (e.g., falls from height, vehicular collisions, electrocution) and suicides (e.g., hanging, carbon monoxide poisoning).

Sample size estimation was performed using PS: Power and Sample Size software (version 3.0) [18]. Based on the prevalence difference reported by Butt et al., a minimum of 23 cases per group (46 in total) was required [19]. After accounting for a 10% buffer for attrition or exclusions, the minimum required sample size was 51 cases overall. However, because a larger pool of eligible cases was available, the final cohort was equally divided to maintain balance, with 51 cases in the CV group and 51 cases in the non-CV group. Cases were selected consecutively during the study period, and assigned to the CV or non-CV group in a 1:1 ratio, based on the availability of suitable cases fulfilling the inclusion and exclusion criteria.

Statistical analysis

All analyses were performed using IBM SPSS Statistics for Windows, Version 29.0 (Released 2022; IBM Corp., Armonk, NY, USA). Demographic data were summarized with descriptive statistics (numbers and percentages). Associations between Frank’s sign and both CV mortality and critical coronary occlusion (≥75% stenosis) were assessed using chi-square tests. A multivariable logistic regression model was applied, with critical occlusion as the dependent variable, Frank’s sign as the primary exposure, and adjustments for age group, sex, and ethnicity.

Ethics approval

This study was approved by the Research Ethics Committee of Universiti Kebangsaan Malaysia, Kuala Lumpur (Reference No.: UKMPPI/111/8/JEP-2023-677).

## Results

A total of 102 cases were selected from 643 postmortem examinations conducted over a 15-month period. The cohort was equally divided into CV deaths (n = 51) and non-CV deaths (n = 51). The mean age was 46.5 years (range, 30-65), with most cases aged 40-59 years (57.8%). Males comprised 83.3% of the cohort. Ethnic distribution was Chinese (55.9%), Malay (30.4%), and Indian (13.7%) (Table 1).

**Table 1:** Descriptive statistics of the study sample (N = 102) SD, Standard Deviation

Parameters	Mean (SD)	Frequency (%)
Age (years)	46.5 (9.9)	-
Age group		
Less than 40 years old	-	30 (29.4%)
40 to 59 years old	-	59 (57.8%)
60 years old and above	-	13 (12.7%)
Sex		
Female	-	17 (16.7%)
Male	-	85 (83.3%)
Ethnicity		
Chinese	-	57 (55.9%)
Malay	-	31 (30.4%)
Indian	-	14 (13.7%)

In the CV group, causes of death included coronary atherosclerosis (n = 22, 43.1%), ischemic heart disease (n = 18, 35.3%), and myocardial infarction (n = 11, 21.6%). In the non-CV group, unnatural deaths accounted for 43 cases (86.3%), and natural deaths for seven cases (13.7%). Traumatic deaths included vehicular crashes and falls (n = 33, 64.7%), suicides (n = 9, 17.6%), and electrocution (n = 2, 3.9%) (Table 2).

**Table 2:** Causes of death in cardiovascular and non-cardiovascular groups (N = 102)

Cause of death	n	% within group
Cardiovascular group (n = 51)		
Coronary atherosclerosis	22	43.1
Ischemic heart disease	18	35.3
Myocardial infarction	11	21.6
Non-cardiovascular group (n = 51)		
Unnatural deaths (total)	44	86.3
Vehicular crashes and falls	33	64.7
Suicides	9	17.6
Electrocution	2	3.9
Natural deaths	7	13.7

Frank’s sign was present in 64.3% of CV deaths, compared with 35.7% of non-CV deaths. This difference was statistically significant (χ²(1, N = 102) = 18.21, p < 0.001), with an odds ratio of 7.8 (95% CI: 2.8-21.5) (Table 3). Similarly, Frank’s sign was observed in 77.1% of cases with critical coronary artery occlusion, compared with 22.9% without occlusion. This association was also statistically significant (χ²(1) = 19.79, p < 0.001), with an odds ratio of 7.4 (95% CI: 2.9-18.9) (Table 3).

**Table 3:** Association between Frank’s sign, cardiovascular death, and critical coronary artery occlusion *p < 0.001, statistically significant; χ², Pearson Chi-Square; CI, Confidence Interval; OR, Odds Ratio

Outcome	Frank’s sign	Yes, n (%)	No, n (%)	χ²	p-value	Odds ratio (95% CI)
Cardiovascular death	Presence	45 (64.3%)	25 (35.7%)	18.21	<0.001*	7.8 (2.8-21.5)
	Absence	6 (18.8%)	26 (81.3%)			
Critical coronary occlusion	Presence	54 (77.1%)	16 (22.9%)	19.79	<0.001*	7.4 (2.9-18.9)
	Absence	10 (31.3%)	22 (68.7%)			

Diagnostic performance metrics are summarized in Table 4. For critical occlusion, sensitivity was 84.4%, specificity 57.9%, PPV 77.1%, and NPV 68.8% (Youden’s index = 0.42). For CV death, sensitivity was 88.2%, and specificity was 51.0% (Youden’s index = 0.39). Youden’s index values (0.39 and 0.42) confirm its moderate discriminative power as a visual indicator of underlying disease.

**Table 4:** Diagnostic performance of Frank's sign in detecting cardiovascular outcomes PPV, Positive Predictive Value; NPV, Negative Predictive Value

Outcomes	Sensitivity (%)	Specificity (%)	PPV (%)	NPV (%)	Youden’s index
Cardiovascular death	88.2	51.0	64.3	81.3	0.39
Critical coronary artery occlusion	84.4	57.9	77.1	68.8	0.42

Multivariable logistic regression confirmed Frank’s sign as an independent predictor after adjustment for age, sex, and ethnicity (AOR = 7.77, 95% CI: 2.71-22.30, p < 0.001), while these demographic variables were not significant predictors (Table 5). Indian ethnicity demonstrated an elevated odds ratio (OR = 4.55, 95% CI: 0.90-23.00), though this did not reach statistical significance. The Hosmer-Lemeshow test indicated good model fit (χ² = 6.64, df = 7, p = 0.468).

**Table 5:** Multivariable logistic regression analysis of categorical predictors for critical coronary artery occlusion Hosmer-Lemeshow test: χ² = 6.64, df = 7, p = 0.468 *p < 0.05, statistically significant; CI, Confidence Interval; OR, Odds Ratio

Predictor variables for critical coronary artery occlusion	p-value	Adjusted OR	95% CI for OR	
			Lower	Upper
Presence of Frank’s sign	<0.001*	7.77	2.71	22.30
Age 40-59 years old	0.606	0.65	0.13	3.28
Age ≥ 60 years old	0.105	3.27	0.78	13.68
Male gender	0.238	0.47	0.14	1.64
Malay vs. others	0.099	3.25	0.80	13.18
Indian vs. others	0.067	4.55	0.90	23.00

## Discussion

This study explored the association between Frank’s sign and CV deaths in a Malaysian autopsy cohort, with a specific focus on its relationship to critical coronary artery occlusion. The findings demonstrate that Frank’s sign is significantly more prevalent in CV deaths compared with non-CV deaths and is an independent predictor of critical coronary artery occlusion.

The study cohort was predominantly male, with a mean age of 46.5 years, and most were of Chinese ethnicity. The male predominance reflects national mortality statistics, where males contribute more frequently to medicolegal autopsies. The relatively young mean age can be explained by the smaller number of cases above 60 years in this series, as medicolegal autopsies are less commonly performed in the elderly age group at this center - particularly those aged 55 years and above - as shown in a previous postmortem study at this center [20]. Another possible explanation for the relatively young mean age is the burden of premature CV mortality in Asia, which is known to have higher premature CV deaths [21]. Previous Frank’s sign studies often reported older cohorts, frequently >60 years [10,15]. 

The population in the catchment area is predominantly Malay, followed by Chinese. The higher proportion of Chinese individuals in this cohort likely reflects referral patterns and case selection within the medicolegal autopsy population, rather than true community demographics [22]. Although Indian ethnicity showed an elevated odds ratio for critical occlusion, the result was not statistically significant, likely due to the small subgroup size. This observation is noteworthy, as prior Malaysian epidemiological studies have consistently shown Indians at the highest risk for premature coronary artery disease [23]. Larger studies are required to determine whether the predictive value of Frank’s sign differs across ethnic groups.

Frank’s sign was present in the majority of CV deaths and cases with critical coronary occlusion. These results support the strong correlation first demonstrated by Kirkham et al. and reaffirmed in later autopsy work [12,14,15]. Importantly, the association remained significant, underscoring the reliability of Frank’s sign as a marker of coronary pathology.

In our cohort, Frank’s sign showed high sensitivity but only moderate specificity. For critical occlusion, sensitivity was 84.4% and specificity was 57.9%; for CV death, sensitivity was 88.2% and specificity was 51.0%. These findings are consistent with Edston, who reported an overall sensitivity of 0.75 and specificity of 0.64 for earlobe crease in relation to coronary artery disease severity [15]. The high sensitivities suggest that the absence of Frank’s sign may be useful in excluding significant coronary disease, but the low specificity (around 50%) substantially limits its confirmatory or diagnostic value in clinical practice. The moderate Youden’s index values in the present study underscore this limitation. Therefore, while Frank’s sign may not be clinically reliable as a diagnostic marker on its own, it may still have supportive value in forensic examinations, where external findings can guide more detailed cardiac dissection.

Multivariable logistic regression confirmed Frank’s sign as an independent predictor of critical coronary occlusion. Age, sex, and ethnicity were not significant predictors in the adjusted model, despite elevated odds ratios in certain subgroups. This finding aligns with previous studies that reported Frank’s sign as the strongest risk factor for coronary artery disease, apart from age and high body mass index [15].

A key strength of this study is the use of autopsy-confirmed causes of death, which eliminates misclassification bias and allows precise pathological determination of coronary stenosis severity. The inclusion of standardized photographic documentation of Frank’s sign further strengthens reproducibility.

This study also has several important limitations. First, the sample size was modest, with underrepresentation of women and individuals aged ≥60 years, leading to wide confidence intervals and limiting subgroup analyses. Second, the study population was drawn from medicolegal autopsies, which may not reflect the living population and restricts generalizability. Third, the non-CV group predominantly comprised unnatural deaths (e.g., vehicular crashes, suicides, electrocution), which typically involve younger, healthier individuals with fewer comorbidities than those dying of coronary artery disease. This demographic imbalance may have exaggerated the observed strength of association between Frank’s sign and CV deaths. Fourth, although multivariable logistic regression was applied, adjustments were limited to age, sex, and ethnicity. Important CV risk factors, including smoking, diabetes, hypertension, and body mass index, were unavailable in autopsy records, and residual confounding cannot be excluded. Finally, assessment of Frank’s sign was based on photographic review, which - although standardized - may still be subject to observer variability.

Despite these limitations, the results highlight the potential of Frank’s sign as a low-cost, non-invasive, and easily recognized marker of coronary atherosclerosis. In forensic practice, its presence during external examination can raise suspicion of underlying CV disease in sudden deaths, guiding a more targeted dissection of the coronary arteries. In clinical practice, it could contribute to the early identification of at-risk individuals, particularly in resource-limited settings.

Future work should include larger multicenter autopsy cohorts with balanced demographic representation and prospective clinical studies correlating Frank’s sign with imaging (computed tomography angiography and echocardiography) and biomarkers. Such studies will help establish whether Frank’s sign has a place in routine CV risk assessment.

## Conclusions

This autopsy-based study supports a significant association between Frank’s sign and CV mortality due to coronary artery atherosclerosis, as well as with critical coronary artery occlusion. While Frank’s sign remained a predictor after adjustment for age, sex, and ethnicity, the absence of information on major CV risk factors (such as smoking, diabetes, hypertension, and body mass index) limits the strength of this conclusion. Taken together, these findings suggest that Frank’s sign may serve as a low-cost and easily recognized supportive or adjunctive marker that raises suspicion of underlying coronary artery disease, particularly in forensic practice. Its clinical utility, however, is limited by low specificity and only moderate discriminative performance, meaning it should not be used as a stand-alone marker. Its predictive value should be interpreted cautiously, and larger multicenter studies with demographically balanced populations and more comprehensive risk factor data are required to validate its clinical and forensic utility.
